# Using Halothermal Time Model to Describe Barley (*Hordeumvulgare* L.) Seed Germination Response to Water Potential and Temperature

**DOI:** 10.3390/life12020209

**Published:** 2022-01-29

**Authors:** Abd Ullah, Sadaf Sadaf, Sami Ullah, Huda Alshaya, Mohammad K. Okla, Yasmeen A. Alwasel, Akash Tariq

**Affiliations:** 1 Xinjiang Key Laboratory of Desert Plant Roots Ecology and Vegetation Restoration, Xinjiang Institute of Ecology and Geography, Chinese Academy of Sciences, Urumqi 830011, China; abdullahbotany123@gmail.com; 2 State Key Laboratory of Desert and Oasis Ecology, Xinjiang Institute of Ecology and Geography, Chinese Academy of Sciences, Urumqi 830011, China; 3 Cele National Station of Observation and Research for Desert-Grassland Ecosystems, Cele 848300, China; 4 University of Chinese Academy of Sciences, Beijing 100049, China; 5 Department of Botany, University of Peshawar, Peshawar 25120, Pakistan; malikanitasadaf@gmail.com; 6 Cell and Molecular Biology, University of Arkansas, Fayetteville, NC 72701, USA; hmalshay@uark.edu; 7 Botany and Microbiology Department, College of Science, King Saud University, P.O. Box 2455, Riyadh 11451, Saudi Arabia; Malokla@ksu.edu.sa (M.K.O.); Yasmeen@ksu.edu.sa (Y.A.A.)

**Keywords:** barley, seed germination, halothermal time model, cardinal temperatures, water potential

## Abstract

Barley (*Hordeum vulgare* L.) is a salt-tolerant crop with considerable economic value in salinity-affected arid and semiarid areas. In the laboratory experiment, the halothermal time (HaloTT) model was used to examine barley seed germination (SG) at six constant cardinal temperatures (Ts) of 15, 20, 25, 30, 35, and 40 °C under five different water potentials (ψs) of 0, −0.5, −1.5, −1.0, and −2.0 MPa. Results showed that at optimum moisture (0 MPa), the highest germination percentage (GP) was recorded at 20 °C and the lowest at 40 °C. Moreover, GP increased with the accelerated aging period (AAP) and significantly (*p* ≤ 0.05) decreased with high *T.* In addition, with a decrease of ψ from 0 to −0.5, −1, 1.5, and −2.0 MPa, GP decreased by 93.33, 76.67, 46.67, and 33.33%, respectively, in comparison with 0 MPa. The maximum halftime constant (θHalo) and coefficient of determination (R2) values were recorded at 20 °C and 30 °C, respectively. The optimum temperature (T_o_) for barley is 20 °C, base Ψ of 50th percentile (Ψb (50)) is −0.23 Mpa, and standard deviation of Ψb (σΨb) is 0.21 MPa. The cardinal *T*s for germination is 15 °C (T_b_), 20 °C (T_o_), and 40 °C (T_c_). The GP, germination rate index (GRI), germination index (GI), coefficient of the velocity of germination (CVG), germination energy (GE), seed vigor index I and II (SVI-I & II), Timson germination index (GI), and root shoot ratio (RSR) were recorded maximum at 0 MPa at 20 °C and minimum at −2.0 MPa at 40 °C. Mean germination time (MGT) and time to 50% germination (T 50%) were maximum at −2 MPa at 40 °C, and minimum at 20 °C, respectively. In conclusion, the HaloTT model accurately predicted the germination time course of barley in response to T, Ψ, or NaCl. Therefore, barley can be regarded as a salt-tolerant plant and suitable for cultivation in arid and semi-arid regions due to its high resistance to salinity.

## 1. Introduction

Barley (*Hordeum* *vulgare* L. Poaceae.) is an important annual cereal crop worldwide and ranks fourth in production [1]. It is used for various purposes, including human food, brewing materials, animal feed, and bedding [2]. Barley can withstand different environmental stress factors such as flood, salinity, and water stress, and it is even more tolerant than wheat under any unsuitable ecological conditions [3]. However, the ability to cope with stress factors largely depends on the intensity of the abiotic stress factors and the stage of the plant’s growth. Likewise, the salt tolerance of barley varies depending on its growth phase as it is most sensitive to salinity during seed germination and the initial stages of seedling development, but as it grows older, it becomes more tolerant tosalinity. Besides, salt stress has been linked to ionic rather than osmotic effects [4,5].

Due to the release of atmospheric CO_2_ and other warming gases, climate change can increase global temperatures by 2.5–4.5 °C [6]. Due to the link between temperature, dormancy, and germination, an increase in temperature canhave a detrimental effect on plant species’ emergence and establishment. Temperature is one of the most important factors influencing seed germination (SG) [7,8,9]. Several plant species undergo seed germination as the first phase of their life cycle [9]. It is a complex physiological process that is affected by environmental stress factors, including temperature (T), water availability, salinity, light, and chemical materials [10,11,12,13,14,15,16]. It has been indicated, however, that temperature and water availability are the main factors influencing SG by affecting seed dormancy, enzymatic activity, hormonal biosynthesis, and translocation of reserve store materials [7,8,17,18]. Furthermore, the ability of the seed to absorb water also determines the success or failure of SG; however, this is determined by soil moisture levels [12].

It has been demonstrated that there are three cardinal temperatures (Ts), which act like three checkpoints that can strictly evaluate the response of SG to temperature in a variety of plant species [12]. They are: (a) The minimum or base temperature (T_b_); SG will cease at Ts below); (b) the optimum temperature (T_o_; SG will commence promptly); and (c) the maximum or ceiling temperature (T_c_); SG will cease at Ts above. However, the Ts may differ among plant species and under different climatic conditions [19]. If, for instance, the increase in temperature exceeds the T_c_ for a given species of plant, then the species will no longer be able to germinate, resulting in a threat to its survival and establishment. Previous studies have reported that the germination rate (GR) increases as the temperature increases between T_b_ and T_o_, but decreases when the temperature increases above T_o_ [11,20,21,22].

In general, GP and GR increase as moisture availability increases but decline under conditions of negative water potential (ψ) [21,23]. Additionally, salt stress affects SG due to osmotic or ion toxicity effects [24,25,26] or a combination of both effects [27]. However, despite the importance of SG under salinity [28,29], the mechanism(s) of salinity tolerance in seeds is still little studied, particularly when compared to the body of knowledge currently available for salinity tolerance in vegetative plants [30,31,32,33,34]. Dissolved salt ions reduce the ψ of salt solutions. For instance, a 1 M NaCl solution has a ψ of −4.4 MPa at 25 °C [35]. 

Moreover, accumulations of Na+ and Cl^−^, as well as osmoprotectants, result in osmotic adjustment or a decrease in ψ inside the seed cells, allowing SG to occur at lower concentrations of ψ [36,37]. Consequently, changes in T, ψ, and salinity influence SG independently and interdependently, affecting the distribution and production of economic crop species [13]. To date, several mathematical models have been developed to illustrate the relationship between SG and T for many crop plants using thermal time models (TT) and hydrotime models (θH) [11,38,39,40]. 

Researchers have previously used a hydro-time model to evaluate the impact of ψ on seed germination at a given temperature [41,42]. According to this model, a negative relationship exists between the time to germination (tg) and the difference between the ψ of the seed environment and the physiological ψ threshold for radicle emergence (base ψ or ψb), which differ among seeds in the population.

In addition, Gummerson’s [41]. hydrothermal time (HTT) model for SG suggests that when Ts *≤* T_o_ (i.e., between T_b_ and T_o_) and at any given ψ, the tg of any germination percentile of a seed population is a function of the degree to which T and ψ exceed their respective base values, T_b_ and ψb, at which germination is hindered [41,43].

Furthermore, one study [38] used the same technique to calculate the SG attributes of the halophyte *Suaeda* *maritime* at different NaCl concentrations at sub-optimal Ts. They proposed a halothermal time (HaloTT) model which substitutes log NaCl for Ψ in the HTT model. Therefore, the germination performance at sub-optimal Ts is shown to be affected by the salinity level threshold distribution (NaCl b(g)) relative to the NaCl of the surrounding environment. In light of increasing global temperatures and salinity, as well as the ever-increasing number of people on earth, it is imperative to study seed germination and early seedling establishment of economic crop plants to abiotic stress factors. Based on its broad stress acclimation, we sought to examine the response characteristics of barley to T, Ψ, and salinity using the HaloTT model. This study aimed to investigate how T and salinity affect the germination features of barely seeds using the HaloTT model and estimate the cardinal Ts and the salt tolerance threshold of barley.

## 2. Methodology

### 2.1. Seeds Germination and Experiment Protocol

The barley (AJJ variety) seeds were generously provided by the Nuclear Institute for Food and Agriculture (NIFA), Peshawar Pakistan. The seeds (95% viability rate) of the same size and shape were surface sterilized with 95% ethanol solution for 3 min and then rinsed with distilled water and shade dried at room temperature [44]. A randomized complete block design (RCBD) Petri dish experiment was performed at the Plant Physiology Laboratory, Department of Botany, University of Peshawar, Khyber Pakhtunkhwa, Pakistan. Halothermal time (HaloTT) experiments were conducted at six constant temperatures of 15, 20, 25, 30, 35, and 40 °C with five water potentials of 0, −0.5, −1.0, −1.5, and −2 MPa. There were ten seeds per Petri dish surface covered with Whatman No. 1 filter paper and three replicates for each treatment. For the stress-treated Petri dishes, 5 mL of NaCl solution was used and for the control Petri dishes, distilled water. ThePetri dishes were placed in an incubator (Memmert Beschickung-Loading-Model 100-800, Schwabach, Germany) in the dark except for the reading times. The seeds were periodically examined and considered to have germinated when the radicle reached a length of 1 mm. In the end, seeds were removed and evaluated for their germination characteristics. The germination time course data were analyzed, and different parameters were determined for the thermal time, halo-time, and halo-thermal time models using repeated probit regression analysis as described previously [45,46]. 

### 2.2. Thermal Time Model (TT)

Sub (TTsub) and supra optimal (TTsupra) cardinal Ts were derived from the formulas based on the halo thermal time model, which is given below,

TT_sub_ = (T − T_b_) t_g_ at sub-optimal *T* (1)

TT_supra_ = (T_c(g) −_ T_b_) t_g_ at supra-optimal *T* (2)

### 2.3. Halotime Model (HT)

The proposed halo-time model (θHalo) is used to enhance model prediction. θHalo determines the connections between solute potential and germination rate in the same manner as the thermal time model does:

θHalo(g) = (NaCl_(g)_ − NaCl_b_) tg (3)

Or

Probit (g) = [NaCl − (θHalo/tg) − NaCl_b_ 50]/σNaCl_b_ (4)

### 2.4. Halothermal Time Model (HaloTT)

For HaloTT model, using the model suggested by Seal et al. [37].

θHaloTT = (NaCl_(g)_ − NaCl_b_) (T − T_b_) tg (5)

Or

Probit (g) = [(NaCl (θHaloTT/(T − T_b_) tg) − NaCl_b_(50)]/σNaCl_b_ (6)

Equation seven is the modified form of equation five for further analysis.

θHaloTT = [NaCl_b(g)_ − NaCl − (kT (T − T_o_))] (T − T_b_) tg (7)

Or

Probit (g) = [NaCl-kT (T − T_o_) − (θHalo/ (T − T_b_) tg) − NaCl_b_(50) ]/σNaCl_b_ (8)

### 2.5. Germination Parameters

The below-mentioned germination indices were calculated from the germination rate, physical observation, seed weight, root and shoot lengths, leaf length, fresh and dry weights of the plants.

#### 2.5.1. Germination Percentage (GP)

GP represents the total number of seeds germinated out of the total seeds sown in each Petri dish. This germination parameter was calculated using the formula [47].

(9) $$\mathrm{Germination}\;\mathrm{percentage}\;(\mathrm{GP})=\frac{\mathrm{Final}\;\mathrm{number}\;\mathrm{of}\;\mathrm{seedlinsg}\;\mathrm{emerged}}{\mathrm{Total}\;\mathrm{number}\;\mathrm{of}\;\mathrm{seeds}\;\mathrm{sown}}\times 100$$

#### 2.5.2. Mean Germination Time (MGT)

The MGT index showed that how fast the seeds emerged in a population. Small MGT value means seed population has a high rate and vice versa. This was calculated using the following formula [47].

(10) $$\mathrm{Mean}\;\mathrm{germination}\;\mathrm{time}\;(\mathrm{MGT})=\frac{\in \mathrm{fx}}{\in f}$$

where f is the number of seeds germinated on day X. 

#### 2.5.3. Germination Rate Index (GRI)

The GRI represents the percent germination on respective days and times. It is calculated by using the following formula [48].

(11) $$\mathrm{Germination}\;\mathrm{rate}\;\mathrm{index}\;(\mathrm{GRI})=\frac{G1}{1}+\frac{G2}{2}+\frac{G3}{3}\ldots .\frac{\mathrm{Gx}}{x}$$

where G_1_ and G_2_ are the percent germinations on the first and second day after sowing and Gx is the final germination percentage on the last day. 

#### 2.5.4. Germination Index (GI)

The germination index tells us about the germination percentage and speed of germination. GI was calculated following the standard methodology [49].

(12) Germination index (GI)=(10×n1)+(9n×n2)…(1n×10)

where n1, n2…n10 showed the frequency of germinated seeds on first, second, and respective days till last day. 

#### 2.5.5. Coefficient of the Velocity of Germination (CVG)

The CVG represents the velocity of germination of seeds in an experiment, which will increase with an upsurge in the frequency of germinated seeds. The highest theoretical CVG value will be obtained when all sown seeds grow on the first day. This is calculated using the formula [50].

(13) $$\mathrm{Coefficient}\;\mathrm{of}\;\mathrm{the}\;\mathrm{velocity}\;\mathrm{of}\;\mathrm{germination}\;(\mathrm{CVG})=\frac{N1+N2+N3\ldots \mathrm{Nx}}{100}\times N1T1\ldots \mathrm{NxTx}$$

in which N is the frequency of seeds germinating every day and T represents the time from sowing to germination of seed N. 

#### 2.5.6. Germination Energy (GE)

Plant germination energy was calculated by using the following formula [51].

(14) $$\mathrm{Germination}\;\mathrm{energy}\;(\mathrm{GE})=\frac{X1}{Y1}+\left(\frac{X2-X1}{Y2}\right)+\left(\frac{\mathrm{Xn}-\mathrm{Xn}-1}{\mathrm{Yn}}\right)$$

Here X_1_, X_2,_ and X_n_ are the frequency of emerged seeds on the first day, second, and so on. While Y_1_, Y_2,_ and Y_n_ are the days from sowing to first, second, and up to last day count.

#### 2.5.7. Timson Germination Index (TGI)

The TGI index represents the average number of seeds germinated per day. This is measured from its mathematical formula as follows [52].

(15) $$\mathrm{Timson}\;\mathrm{germination}\;\mathrm{index}\;(\mathrm{TGI})=\frac{\mathrm{ti}+\left(N/2-\mathrm{ni}\right)\left(\mathrm{tj}-\mathrm{ti}\right)}{\left(\mathrm{nj}-\mathrm{ni}\right)}$$

where G is the total percentage of germination per day and T time of germination.

#### 2.5.8. Mean Germination Rate (MGR)

Mean germination rate is the reciprocal of mean germination time. It was found out through the following formula [53].

(16) $$\mathrm{Mean}\;\mathrm{germination}\;\mathrm{rate}\;(\mathrm{MGR})=\frac{1}{\mathrm{Mean}\;\mathrm{Germination}\;\mathrm{Time}}$$

#### 2.5.9. Seed Vigor Index-I (SVI-I) 

The length of three seedlings from each pot was measured in cm and then calculated in the following formula [54].

(17) Seed Vigor Index=Seedlings length(cm)×Seed Germination %age

#### 2.5.10. Seed Vigor Index-II (SVI-II)

The dry weight of three seedlings from each pot was determined through electrical balance. The values were put in the formula and multipliedwith seed germination percentage. The formula is as follows [55].

(18) Seed Vigor Index=Seed dry weight (mg)×Seed Germintion

#### 2.5.11. Time to 50% Germination (T50%)

This index was developed to find out the time required for 50% seed germination. This is reported through the following mathematical formula [56].

(19) $$\mathrm{Time}\;\mathrm{to}\;50\%\;\mathrm{germination}\;(T50\%)=\frac{\mathrm{ti}+\left(N/2-\mathrm{ni}\right)\left(\mathrm{tj}-\mathrm{ti}\right)}{\left(\mathrm{nj}-\mathrm{ni}\right)}$$

where N final number of seeds emerged, nj and ni are the cumulative numbers of seeds emerged after adjacent counts during tj and ti, when ni < N/2 > Nj.

#### 2.5.12. Root-Shoot Ratio (RSR) 

The RSR ratio was recorded after the root and shoot were dried in an oven for 24 h. Which is then calculated through the following formula [53].

(20) $$\mathrm{Root}-\mathrm{shoot}\;\mathrm{ratio}=\frac{\mathrm{root}\;\mathrm{dry}\;\mathrm{weight}}{\mathrm{shoot}\;\mathrm{dry}\;\mathrm{weight}}.$$

### 2.6. Data Analysis

The investigation of temperatures (thermal time), water potentials (halftime), and their interactions (halothermal time model) on seed germination rate and germination attributes were analyzed through analysis of variance (ANOVA) using IBM SPSS Statistics 26. ANOVA was applied using three replicates of each treatment and germination parameters. The basic statistical calculation was performed in excel software. The values of the following parameters: Ψ_b(50)_, σΨ_b_; *R*^2^, Sig, and F were determined using linear probit regression analysis in SPSS. ORIGIN2021 PC Corporation was used for plotting various graphs of germination fraction vs. accelerated aging period and germination parameters against T and Ψ.

## 3. Results

**Figure 1:**
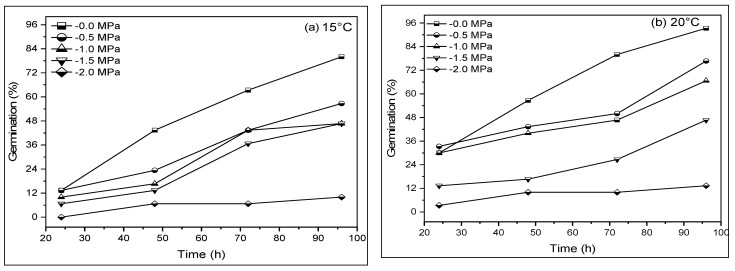

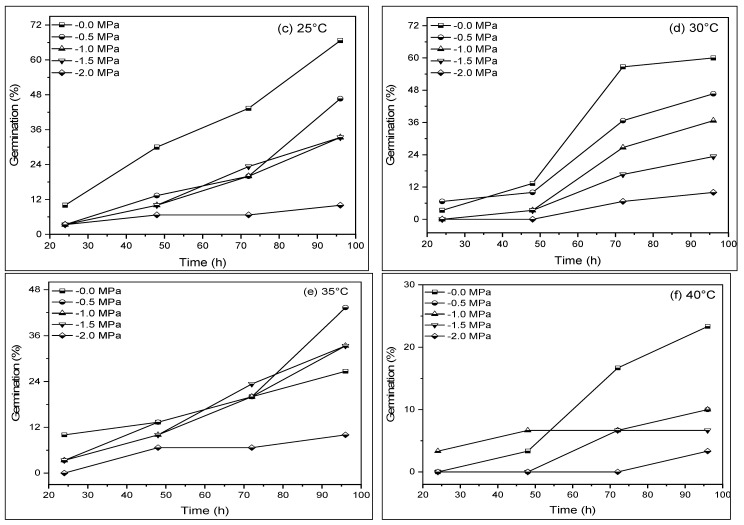
Cumulative germination for *Hordeum vulgare* L. at (**a**) 15 °C, (**b**) 20 °C, (**c**) 25 °C, (**d**) 30 °C, (**e**) 35 °C, and (**f**) 40 °C having different water potentials. Symbols indicate water potential and lines indicate cumulative germination.

The water potential (Ψ), temperature (T), and their interaction (T×Ψ) significantly (*p* ≤ 0.05) affected germination percentage (GP) and germination rate (GR) (Figure 1). A maximum GP was recorded at 20 °C and a minimum at 40 °C at optimum moisture (0 MPa; control). Accordingly, minimum germination of 3.33% was reported at 40 °C under −2.0 MPa and a maximum of 76.67% at 20 °C under −0.5 MPa compared to the control (0 MPa). This indicates that GP reduced with the reduction in Ψ at each T (Figure 1a–f). Moreover, GP was recorded at a maximum after the fourth day at 0 MPa. GP generally increased with accelerated aging (AAP) and decreased significantly (*p* < 0.05) with high T. Further, with the decrease ofψfrom 0 to −0.5, −1, 1.5, and −2.0 MPa, GP decreased by 93.33, 76.67, 46.67, and 33.33% in comparison with the control (0 MPa), respectively (average for all levels of AAP) (Figure 1b).

**Figure 2:**
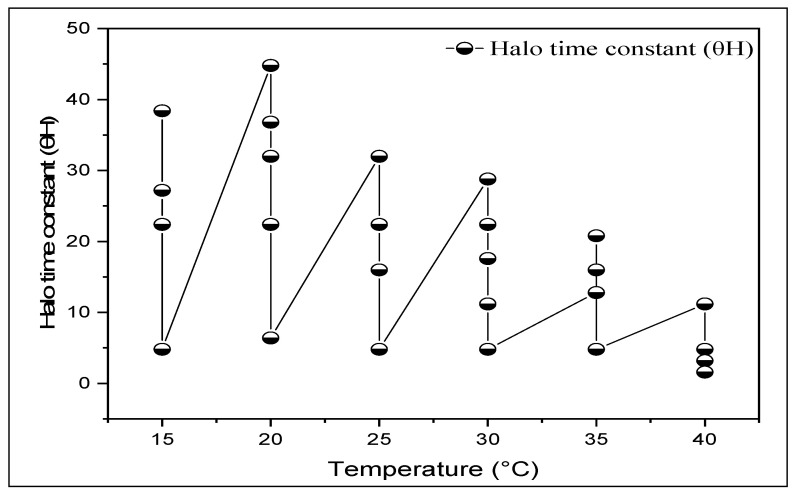
Plot showing changes in halotime constant (θH) as a function of temperature (T) for *Hordeum vulgare* L.

The maximum halothermal time constant (*θ*Halo) and *R*2 values were recorded at 20 °C and 30 °C, respectively (Table 1, Figure 2). Compared with 0 MPa, the highest TTsub and TTsupra values were observed at 20 °C at −0.5 MPa and decreased with decreasing Ψ (−2.0 MPa). In addition, GR (g) values show a significant (*p* ≤ 0.01) increase with the decrease inΨ(more negative) at all *T*s (Table 1). The standard deviation σψb values presented comparatively fewer fluctuations at all *T*s. The highest σψb was recorded at 20 °C, and lowest was recorded at 40 °C (Table 2). Similarly, the highest base water potential at 50% germination (ψb(50)) value was observed at 40 °C (−2.0 MPa). Seeds exhibit a base or minimum temperature (T_b_) below which germination is decreased, an optimum temperature (T_o_) at which germination is most rapid, and a maximum or ceiling temperature (T_c_) at which germination is prevented. Germination at suboptimalTcan be characterized based on thermal time, or theTin excess of T_b_ multiplied by the time to a given germination percentage (tg).

The minimum temperature (T_b_) for barley observed from our experiment is 15 °C, below which the germination rate decreases, and it will become difficult for a plant to continue its physiological processes (Table 3). The optimum temperature (T_o_) at which barley germination was maximum was 20 °C. The maximum or ceilingtemperature (T_c_) above which plants cannot continue their physiological and biochemical activities was 40 °C. 

**Figure 3:**
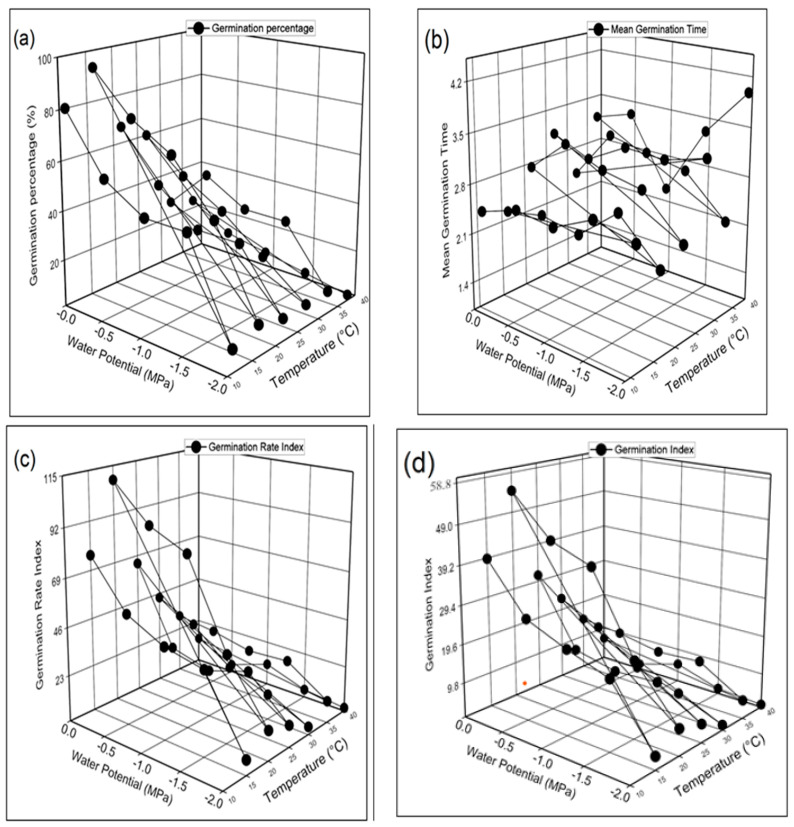
Interactive effect of salinity and temperature on (**a**) germination percentage (**b**) mean germination time (**c**) germination rate index (**d**) germination index of barley (*Hordeum* *vulgare* L.) var. AAJ using halo thermal time (HaloTT) model.

**Figure 4:**
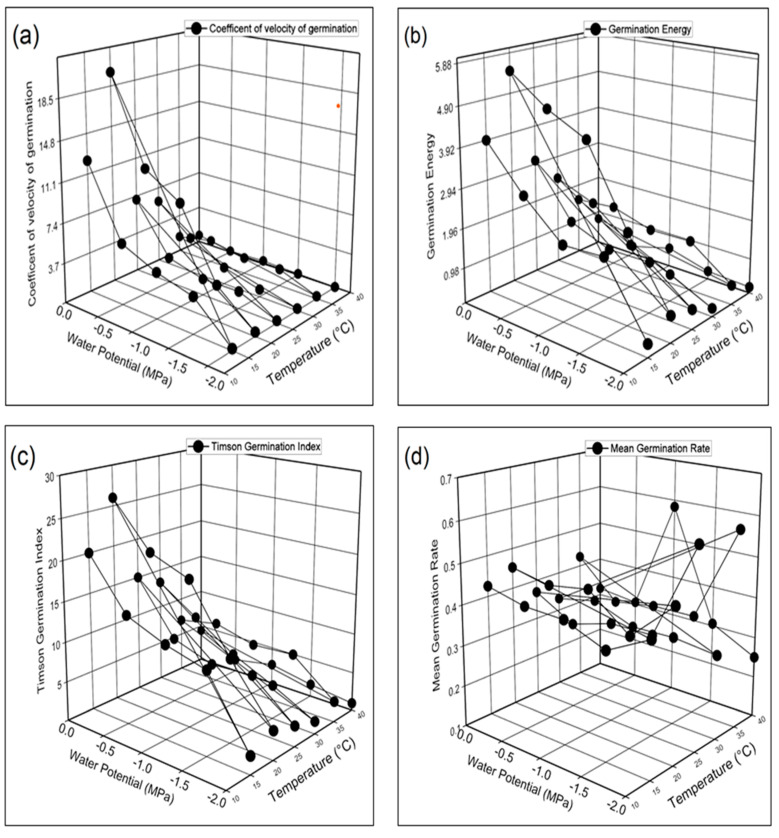
Interactive effect of salinity and temperature on (**a**) coefficient of the velocity of germination (**b**) germination energy (**c**) Timson germination index (**d**) mean germination rate of barley (*Hordeum* *vulgare* L.) var. AAJ using HaloTT model.

**Figure 5:**
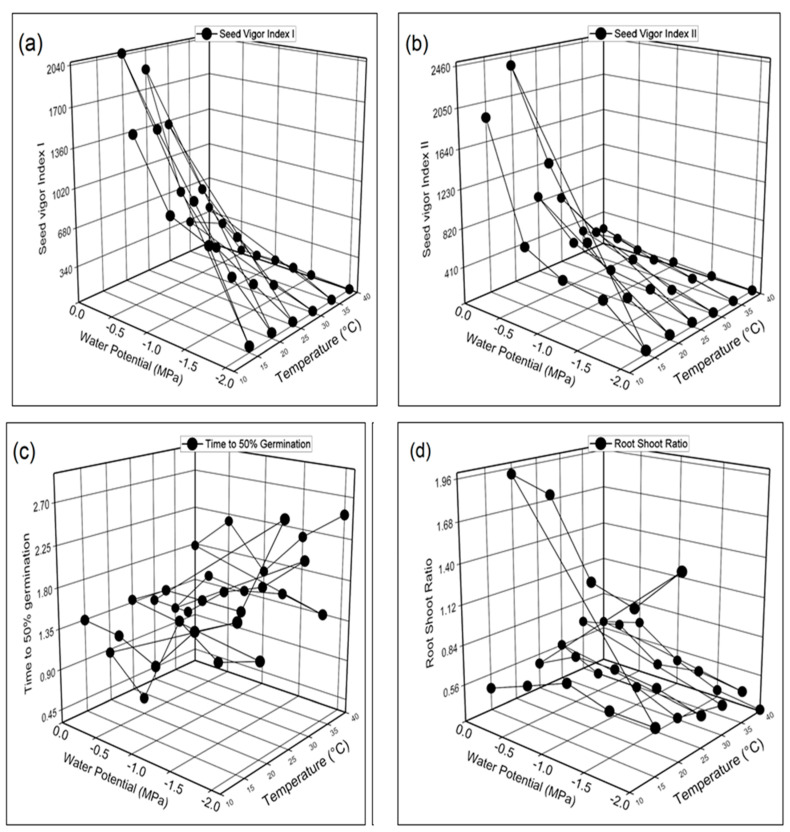
Interactive effect of salinity and temperature on (**a**) seed vigor index-I (**b**) seed vigor index-II (**c**) time to 50% germination (**d**) root shoot ratio of barley (*Hordeum* *vulgare* L.) var. AAJ using HaloTT model.

The results obtained from the recent HaloTT experiment revealed that temperature and NaCl significantly influenced the germination parameters of barley (*Hordeum* *vulgare* L.). The germination percentage (GP), germination rate index (GRI), and germination index (GI) were maximum in seeds grown at 20 °C in 0 MPa distilled water and minimum at 40 °C in −2.0 MPa (Figure 3a–d). Mean germination time (MGT) was recorded maximum at 40 °C in −2.0 MPa and minimum at 20 °C in −2.0 MPa. Figure 4a–d demonstrates the highest and lowest coefficient of the velocity of germination (CVG), germination energy (GE), Timson germination index (TGI), and mean germination rate (MGR) were recorded at 20 °C and 40 °C in 0 MPa and −2.0 MPa respectively. Figure 5a–d shows that seed vigor index I (SVI-I), seed vigor index II (SVI-II), and root shoot ratio (RSR) were recorded as maximum in distilled water (0 MPa) at 20 °C while the minimum is −2.0 MPa at 40 °C. The maximum value of time to 50% germination (T 50%) was recorded in −2.0 MPa at 40 °C, and the minimum value was recorded at 20 °C.

## 4. Discussion 

The HTT model, also known as population-based threshold models, accurately reflects the observed responses of GRs and GPs to stress, age, hormones, and other factors that affect seed germination. An investigation of seed germination under varying environmental conditions will better understand germination parameters and identify the most appropriate geographic location for a species to emerge and establish [11]. The results of the current halothermal time model revealed that water potential (Ψ), temperature (T), and their interaction (T × Ψ) significantly affect the germination rate and germination percentage. In comparison with control (0 MPa), the minimum germination was reported at 40 °C under −2.0 MPa, while the maximum was reported at 20 °C under −0.5 MPa, indicating that GP decreased as T decreased (Figure 1a–f). These findings are in agreement with the previous studies that have indicated that temperature is a critical factor that negatively impacts seed germination by influencing the GP and GR in a wide range of plant species [16,18,57]. In the present study, GP increased with the accelerated aging period (AAP) and significantly (*p* ≤ 0.05) decreased with high temperature. Additionally, stress related to Ψ is another major environmental factor that limits SG and the early establishment of seedlings [39,58,59]. Furthermore, when Ψ is reduced from 0 to −0.5, −1, 1.5, and −2 Mpa, GP decreases by 93.33, 76.67, 46.67, and 33.33%, respectively, as compared to the control (0 Mpa) (average for all levels of AAP) (Figure 1b). Previous findings also reported that the longer AAP and lower ψ (more negative) decreased GP and GR in various crop species [40,43]. 

Further, the θH values increased with an increase in cardinal Ts up to T_o_ and then linearly decreased with a decrease in T > T_o_. Previous studies have also demonstrated that the values of θH have increased at suboptimal temperatures for potato [57] and watermelon [12,40].

Similarly, the results of the base water potential at the 50th percentile (ψb (50)) have also been shown to increase (become more positive) at supraoptimal temperatures [20,23]. In a previous study [16] both GP and GR decreased with decreasingψand increasing NaCl at each tested T. 

In the present study, we found that the GR(g) values increased significantly (*p* ≤ 0.01) with decreasing Ψ (more negative) at all cardinal temperatures Ts (Table 1). GR decreased when water potential was reduced compared to the control. The effect of ψ on GP and GR was greater than that of AAP [42]. In the present study, the minimum temperature (T_b_) for the studied plants was 15 °C, below which the GR decreased. In addition, the optimum temperature (T_o_) for barley germination was 20 °C, whereas the limiting temperature (T_c_) beyond which plants could not continue their physiological and biochemical activities was 40 °C. The previous finding also observed that the temperature spectrum for seed germination contains three cardinal temperatures (Ts), which are crucial in determining the seed germination characteristics [12,19].

Further, we found that the germination percentage and other characteristics were maximum at 0 Mpa (distilled water) at 20 °C, while the minimum was obtained at −2.0 Mpa at 40 °C. The maximum time to 50% germination and mean germination time was observed in −2.0 Mpa at 40 °C, and the minimum value was recorded at 20 °C (Figure 3a–d, Figure 4a–d, and Figure 5a–d). It has also been reported previously that temperature is a key factor that affects both GP and the GR [12,18,60,61]. Moreover, salinity may limit SG by osmotic and ion-specific mechanisms [24,25]. Salinity, temperature (T), and water potential (ψ) all influence SG independently and together. The reason for this may be a result of Na^+^ and Cl^−^ ions entering into seed cells, reducing their osmotic potential and increasing embryonic turgor, which permits the seeds to germinate at lower ψ *s* [16].

## 5. Conclusions

In the present study, the best T and Ψ for barley are 20 °C and 0.0 Mpa, which indicates that GP and GR are significantly affected by T, Ψ, and their interactions with accelerated aging periods. The maximum θHalo and *R*2 values were recorded at 20 °C and 30 °C, respectively. Further, Ψb (50) is −0.23 MPa and σΨb is 0.21 MPa at kT 0.104 MPa. For barley, the cardinal temperatures are recorded as T_b_ = 15 °C, T_o_ = 20 °C, and T_c_ = 40 °C. Hence, the halothermal time (HaloTT) model accurately predicted the germination time course of Barley (*Hordeum* *vulgare* L.) in response to various regimes of T, Ψ, or NaCl. Even though actual scenarios can vary according to field conditions, performance may not exactly follow the predicted model. Yet, a recent study [62] used the HTT model and concluded that the germination sensitivity to Tand ψ of 13 native desert annual plant species were highly correlated with seedling emergence in Arizona’s actual desert field conditions. Accordingly, we believe that the HaloTT model developed in this study can quantify and predict the relative germination attributes expected in the actual field conditions.

## Figures and Tables

**Table 1:** The estimated parameters of the halo and thermal time model to describe *Hordeum* *vulgare* L. seed germination under different temperatures (Ts) and water potentials (ψs).

T	Ψ (MPa)	TTsub (θT1)	TTsupra (θT2)	θ_Halo_ (MPa h)	θHaloTT (MPa h)	Halo Time GR_(g)_	Thermal Time GR_(g)_
15 °C	0	768	1920	38.4	384	0.013	0.013
	−0.5	544	1360	27.2	272	0.018	0.018
	−1.0	448	1120	22.4	224	0.022	0.022
	−1.5	448	1120	22.4	224	0.022	0.022
	−2.0	96.0	240.0	4.8	48.0	0.104	0.104
20 °C	0	896	2240	44.8	448	0.011	0.011
	−0.5	736	1840	36.8	368	0.014	0.014
	−1.0	640	1600	32.0	320	0.016	0.016
	−1.5	448	1120	22.4	224	0.022	0.022
	−2.0	128	320.0	6.40	64.0	0.078	0.078
25 °C	0	640	1600	32.0	320	0.016	0.016
	−0.5	448	1120	22.4	224	0.022	0.022
	−1.0	320	800.0	16.0	160	0.031	0.031
	−1.5	320	800.0	16.0	160	0.031	0.031
	−2.0	96.0	240.0	4.80	48.0	0.104	0.104
30 °C	0	576	1440	28.8	288	0.017	0.017
	−0.5	448	1120	22.4	224	0.022	0.022
	−1.0	352	880.0	17.6	176	0.028	0.028
	−1.5	224	560.0	11.2	112	0.045	0.045
	−2.0	96.0	240.0	4.80	48.0	0.104	0.104
35 °C	0	256	640.0	12.8	128	0.039	0.039
	−0.5	416	1040	20.8	208	0.024	0.024
	−1.0	320	800.0	16.0	160	0.031	0.031
	−1.5	320	800.0	16.0	160	0.031	0.031
	−2.0	96.0	240.0	4.80	48.0	0.104	0.104
40 °C	0	224	560.0	11.2	112	0.045	0.045
	−0.5	96.0	240.0	4.80	48.0	0.104	0.104
	−1	96.0	240.0	4.80	48.0	0.104	0.104
	−1.5	64.0	160.0	3.20	32.0	0.156	0.156
	−2.0	32.0	80.0	1.60	16.0	0.313	0.313

Temperatures (T); water potential (ψ); thermal time constant at sub-optimal temperature (TTsub); thermal time constant at supra-optimal temperature (TTsupra); halotime constant (θH); halothermal time constant (θHTT); germination rate (GR).

**Table 2:** Predictable values of R^2^, ψ_b(50)_ and σψ_b_ using halo thermal time (HaloTT) model to describe *Hordeum* *vulgare* L. seed germination under different Ts and **ψ**s.

Barley	T(°C)	ψ_b(50)_ (MPa)	σψ_b_ (MPa)	R	R^2^	T	Sig.
	15	−0.20	0.13	0.940	0.884	18.47	Sig
	20	−0.23	0.21	0.976	0.953	19.42	0.018
AAJ	25	−0.17	0.12	0.965	0.930	13.36	0.004
	30	−0.13	0.9	0.999	0.998	27.78	0.008
	35	−0.10	0.8	0.555	0.308	7.161	0.000
	40	−0.8	0.6	0.901	0.812	11.64	0.331

R and R^2^ is the coefficient determination, σψb is the standard deviation. Ψb(50) is base water potential at 50 percentiles, θH is halotime constant, F is variability between different means, Sig. is significant value.

**Table 3:** Estimated values of k_T_, σψ_b_, and T_o_ using halothermal time model (HaloTT) for describing seed germination of *Hordeum* *vulgare* L. under different Ts and ψ*s*.

Variables	*Hordeumvulgare* L.
Halothermal time model parameters	
ψ_b(50)_ (MPa)	−0.23
σψ_b_ (MPa)	0.21
θHalo (MPa °C h^−1^)	17.65
k_T_ (MPa °C h^−1^)	0.104
Cardinal temperatures	
T_b_ (°C)	15
T_o_ (°C)	20
T_c_ (°C)	40
R^2^	0.953

Ψ_b(50)_ = base water potential at 50 percentiles, T_b_ = base temperature, T_o_ = optimum temperature, T_c_ = ceiling temperature.

## Data Availability

All the data are available in the manuscript.
