# Correction: Ullah et al. Using Halothermal Time Model to Describe Barley (*Hordeum vulgare* L.) Seed Germination Response to Water Potential and Temperature. *Life* 2022, *12*, 209

**DOI:** 10.3390/life16020267

**Published:** 2026-02-04

**Authors:** Abd Ullah, Sadaf Sadaf, Sami Ullah, Huda Alshaya, Mohammad K. Okla, Yasmeen A. Alwasel, Akash Tariq

**Affiliations:** 1 Xinjiang Key Laboratory of Desert Plant Roots Ecology and Vegetation Restoration, Xinjiang Institute of Ecology and Geography, Chinese Academy of Sciences, Urumqi 830011, China; 2 State Key Laboratory of Desert and Oasis Ecology, Xinjiang Institute of Ecology and Geography, Chinese Academy of Sciences, Urumqi 830011, China; 3 Cele National Station of Observation and Research for Desert-Grassland Ecosystems, Cele 848300, China; 4 University of Chinese Academy of Sciences, Beijing 100049, China; 5 Department of Botany, University of Peshawar, Peshawar 25120, Pakistan; 6 Cell and Molecular Biology, University of Arkansas, Fayetteville, NC 72701, USA; 7 Botany and Microbiology Department, College of Science, King Saud University, P.O. Box 2455, Riyadh 11451, Saudi Arabiayasmeen@ksu.edu.sa (Y.A.A.)

## Error in Title

In the original publication [1], there was an error in the title regarding the species name. The correct title should be:

“Using Halothermal Time Model to Describe Barley (*Hordeum vulgare* L.) Seed Germination Response to Water Potential and Temperature”.

## Text Correction

There was an error in the original publication [1]. The Equations (15) and (18) of Timson germination index (TGI) and the time for 50% germination are both the same, and also the remark provided for Equation (15) does not follow the equation. A correction has been made to Section 2.5.7, “Timson Germination Index (TGI)” and Section 2.5.10, “Seed Vigor Index-II (SVI-II)”:

### *2.5.7. Timson Germination Index (TGI)* 

The Timson Germination Index (TGI) quantifies germination speed using the following formula [52]:

(15) Timson Germination Index (TGI)= ΣG/t

where G is the number of seeds germinated on a given day and t is the corresponding day number (e.g., 1, 2, 3…).

### *2.5.10. Seed Vigor Index-II (SVI-II)* 

The dry weight of three seedlings from each pot was measured with a precision balance. These measures were used to calculate the Seed Vigor Index according to the following formula [55]:

(18) $$\mathrm{Seed}\;\mathrm{Vigor}\;\mathrm{Index}\;=\;Seed\;dry\;weight\;\left(mg\right)\times Seed\;Germintion\;\%$$

The authors state that the scientific conclusions are unaffected. This correction was approved by the Academic Editor. The original publication has also been updated.
